# Avoiding Health Pitfalls of Home Energy-Efficiency Retrofits

**DOI:** 10.1289/ehp.119-a76

**Published:** 2011-02

**Authors:** John Manuel

**Affiliations:** **John Manuel** of Durham, NC, is a regular contributor to *EHP* and the author of *The Natural Traveler Along North Carolina’s Coast* and *The Canoeist*

Housing consumes 40% of our nation’s energy use,^1^ making it a prime target for energy-efficiency measures. Steps such as adding insulation, installing high-efficiency HVAC (heating, ventilation, and air-conditioning) systems, and tuning furnaces rank high as simple ways to lower utility bills and improve comfort and indoor air quality. But an energy-efficiency label attached to a product is meaningless if that product is installed incorrectly, and when it comes to green building techniques, the devil is in the details. The complexities of high-tech equipment and the subtle and usually invisible movement of air and moisture in homes mean even experienced and well-intentioned contractors do not get things right in every instance. This can result in health problems for occupants and installers alike.

Such concerns have arisen in relation to recent activities conducted by local community action agencies through state programs funded by the U.S. Department of Energy (DOE) Weatherization Assistance Program. This program, created under the Energy Conservation and Production Act of 1976, provides the means for basic weatherization of the homes of low-income families. Since 2000, federal funding for the Weatherization Assistance Program has averaged around $225 million per year,^2^ sufficient to weatherize approximately 95,000 homes annually.^3^

In 2009, as part of the American Recovery and Reinvestment Act (ARRA), the federal government awarded the states $5 billion with the goal of weatherizing 600,000 homes by 2012.^4^ But the sudden influx of cash and the short period of time in which to spend it has spelled trouble for many state weatherization programs.

## Missed Steps

No health problems are reported to have resulted from ARRA-subsidized energy-efficiency retrofit activities. But inspections have uncovered several instances of hazardous conditions created or worsened by retrofits, which serve as reminders of the need for care to ensure that home renovations don’t cause more problems than they cure.

For example, in Cook County, Illinois, 12 of 15 homes audited by the DOE Inspector General after receiving retrofits were found to have substandard work, and 5 of 6 furnace tune-ups had not been correctly performed, allowing the heating systems to either improperly fire or exceed maximum allowable carbon monoxide (CO) emissions.^5^ CO is a colorless and odorless gas that, if drawn into the living space of a home, can sicken or kill the occupants. The Centers for Disease Control and Prevention reports that non-fire-related CO poisoning results in an estimated 15,000 emergency room visits and 500 unintentional deaths in the United States per year.^6^

A similar review conducted in Nueces County, Texas, showed the community action agency performing weatherization under ARRA failed to install or document installation of CO detectors in 11 homes inspected, a requirement for any unit with a combustion appliance. The agency also failed to administer and/or document required CO testing of combustion appliances in each of 13 homes inspected. Appliances in 5 of these homes were later found to exceed CO emission allowances.^7^

In Alaska the Fairbanks *Daily News-Miner* reported mold cropping up in houses that had been recently weatherized, explaining, “Homes in cold climates are susceptible to mold because of the extreme temperature differential between inside and outside. Mold needs water to grow, and moisture develops in homes when water vapor inside hits cold surfaces such as windows and outdoor walls and condenses into liquid.”^8^ Evidence to date suggests mold spores in indoor air can cause asthma symptoms, respiratory infections, and upper respiratory problems among susceptible persons.^9^

Critics say problems of poor workmanship in state weatherization programs over the past year are often the result of the programs’ hiring of large numbers of new contractors, not all of whom are properly trained or supervised. In Illinois, for example, the weatherization agency’s pool of contractors grew from 18 to 60 companies to accommodate the increase in production resulting from the infusion of ARRA money.^5^ In a memorandum to the Assistant Secretary of Energy Efficiency and Renewable Energy, DOE inspector general Gregory Friedman stated, “The weatherization contractor and local level inspection deficiencies, in our opinion, raise concerns regarding the adequacy of training and adherence to standards designed to ensure quality workmanship.”^10^

Problems with home energy retrofits are not limited to fly-by-night contractors. Even experienced contractors can be challenged when trying to deliver a product that is at once energy efficient and healthy. Houses are complex systems. The shell (walls, floors, ceiling), HVAC system, and ductwork all interact with each other—change one, and you may inadvertently affect another.

## Tight Squeeze

Tightening houses—that is, reducing the amount of outside air being pulled into the living space and heated or cooled air leaking out—is one of the principal goals of weatherization. However, it is important not to make a building too tight. The American Society of Heating, Refrigerating, and Air-Conditioning Engineers (ASHRAE) publishes a standard that lists recommended levels of fresh-air ventilation for different types of buildings or rooms within buildings, taking into account the size of the space, number of occupants, and use of the space.^11^ These levels reflect optimal energy usage, comfort, and health and should be followed by contractors anytime an extensive retrofit of a building is undertaken.

Tightening a building to the point where it actually reduces energy consumption is relatively easy in new construction; it is more difficult in an existing building because sources of air infiltration may be difficult to find and access. Measures such as adding storm windows and weatherstripping doors may improve comfort, but experts say they have a minimal effect on energy bills. “Air escapes from the top of a building, not the sides,” says Arnie Katz, senior building science consultant with Advanced Energy Corporation, a North Carolina–based energy-efficiency services firm. “If you just tighten the sides, all you are doing is creating a more efficient cylinder.”

Katz says the first priority in tightening a building should be to seal leaks in the top; the second priority is sealing the bottom. Air, he explains, typically leaks from the home through gaps around wiring, plumbing, ceiling lights, vents, and fans.

These gaps are often difficult to access, and workers trying to reach them may find themselves facedown in fiberglass insulation, so it is important they wear proper clothing and face masks when doing this work. The Occupational Safety and Health Administration (OSHA) advises that fibers freed from insulation can cause, skin, eye, and respiratory irritation, and requires that employers provide workers with proper respiratory protection when they are blowing fiberglass insulation into an attic.^12^

Assuming one can actually seal these leaks, the other challenge in tightening homes is to do so without compromising the occupants’ health. This is especially a concern for buildings with indoor combustion appliances such as oil, gas, or propane furnaces and water heaters, which can produce CO. Katz warns, “Anything you do to make a home tighter has the potential for causing backdrafting^13^ of these appliances, because you are changing the air pressure relationships inside the house. That’s why it’s so important to hire someone who knows what they’re doing.”

Trapping of radon is another potential result of overtightening a home. This radioactive gas found in soil, rock, and water can enter the home through dirt crawlspaces and cracks in basement floors and foundations.^14^ Long-term exposure to radon is estimated to cause approximately 20,000 cancer deaths per year.^14^ Homeowners can easily test for radon with a simple kit that can be purchased at home-improvement stores for about $10–15. If detected, radon usually can be vented to the outdoors by installing vent pipes in the crawlspace or basement.

Moisture from outside air, showers, cooking, plumbing leaks, and human activity is another variable that must be dealt with when weatherizing a home. Tightening a house can close off avenues where moisture previously escaped, so it is important to identify how and where moisture is generated and how it can be controlled. Some moisture in the air is desirable, but as with ventilation, a balance must be struck, because over time, excessive moisture can contribute to asthma and allergies and can increase the presence of mold, dust mites, and vermin.^15^

## Getting It Right

Moisture generated in bathrooms and kitchens can be addressed by adding or upgrading fan systems. ASHRAE recommends fans for kitchens and baths that circulate a minimum of 50 cubic feet per minute (cfm). However, Katz cautions the manufacturer’s rating on a fan may not be what you get when it is installed in a home. “The rating is based on a fan sitting on a table and attached to a straight ten-foot run of duct,” he says. “In a house, the fan may be connected to thirty feet of duct that turns at a right angle before exiting outdoors.” In that case, he says, you would need a fan rated at 70 cfm to achieve a 50-cfm result—a judgment that can be made by an experienced builder.

Sealing ductwork is widely considered one of the best ways to not only save energy but also improve indoor air quality. When the blower in an air handler is activated, it creates negative air pressure in the return air ducts. Leaky duct systems that pass through unsealed crawlspaces and attics suck in outside air—which may include dustborne metals, pollen, pesticides, particulates, and mold spores—and redistribute it throughout the home. Government sources say sealing ducts can reduce energy consumption by as much as 20% and reduce the amount of air pollutants redistributed indoors.^16^

In addition to sealing ductwork, DOE and many building experts now recommend sealing vented crawlspaces, especially in areas of high relative humidity.^17^ Homes in the U.S. Southeast historically have been built over vented crawlspaces with the idea that it is essential to circulate cooler air under the floor in summer and to allow moisture that emanates from the soil a way to disperse. However, building scientists have now determined that allowing humid outside air to enter crawlspaces can cause moisture and mold problems. “Venting crawlspaces made sense only when you had no air conditioning, no insulation, and no crawlspace walls,” says Joe Lstiburek, a professional engineer and partner at Boston-based Building Science Corporation.

Lstiburek and some other energy experts also recommend sealing vented attics—another move that would have been considered heresy just a decade ago. The rationale for venting attics has primarily been to flush the heat that radiates through the roof in summer. But by insulating the underside of the roof instead of the ceiling and closing off the gable and soffit vents at the ends of the roof and under the eaves, temperatures in the attic are reduced as less heat radiates through the roof and hot exterior air is barred from coming in the vents. “We’ve seen summer temperatures [in unvented attics] dropping from 140 to 85 degrees,” says Ed Reeves, engineering manager for insulation manufacturer Icynene Corporation. This means HVAC systems can operate more efficiently, as air leaking or radiating from the system is contained in the conditioned space, and systems are not exposed to temperature extremes.

One of the most efficient ways to insulate a roof, for both new and existing homes, is a product known as spray foam insulation. As its name implies, spray foam is sprayed from a gun and readily sticks to most surfaces, expanding to provide a highly effective seal. Reeves says medium-density spray foam has a higher R-value (i.e., insulating value) per inch than fiberglass or cellulose. But of course, Katz says, as with everything else, spray foams can be (and sometimes are) poorly installed and therefore don’t always perform as advertised.

Spray foam does have its disadvantages. It can cost more than four times as much as fiberglass or cellulose, installed, and it can be a nuisance to install. Michael Chandler of North Carolina–based Chandler Design-Build describes what can happen if proper precautions are not taken: “As the foam is sprayed, small droplets of foam end up in the air. This stuff gets in your hair, in your skin, and on your clothes.”

Moreover, although there are no published reports of health effects resulting directly from spray foam, the product does contain isocyanates, and unprotected exposure to this class of chemicals may cause asthma, lung damage, respiratory difficulties, and eye and skin irritation.^18^ Spray foams also contain the flame retardant 1,2,3-trichloropropane (TCP). This chemical is not known to be hazardous to humans when used as intended, but poisoning can occur with acute overexposure,^19^ and there is evidence it causes cancer and liver and kidney damage in rodents.^20^ TCP also is drawing more attention because of increased recognition of its occurrence in groundwater and its resistance to natural degradation.^21^

Professional installers wear personal protective equipment to prevent exposure to foam droplets, Reeves says. But Chandler says that is not always true of workers who come in after the spray foam has dried to shave it down to a flat surface. Despite industry recommendations, “the people doing the shaving often don’t even wear a dust mask,” Chandler says.

## Toward Better Retrofits

To ensure energy-efficiency retrofits provide a net benefit for occupants, the DOE is developing a set of voluntary guidelines for workers involved with weatherization assistance and home energy upgrade program activities.^22^ The guidelines will outline steps and specifications to ensure various types of retrofit jobs are conducted properly as well as essential skills contractors must have to perform the work. The guidelines are expected to help state weatherization programs choose qualified contractors to perform the work in homes. They also will help trainers provide appropriate content for an energy-efficiency retrofit workforce that is expected to grow in coming years.

In a related project, the EPA has drafted complementary guidelines that focus specifically on health and safety of workers and occupants in conjunction with energy-efficiency upgrades.^23^ Once finalized, these guidelines will provide steps contractors can take to ensure retrofit activities do not introduce hazards as well as detect and correct any indoor air quality issues that do arise during work. The EPA also plans to publish sample assessment tools for inspectors and contractors.

In the meantime, says Keith Aldridge, vice president of building science with Advanced Energy, the best way for consumers to ensure they get quality work is to search online for discussions about retrofit-related products and rely on word of mouth for advice on contractors.

## Figures and Tables

**Figure:**
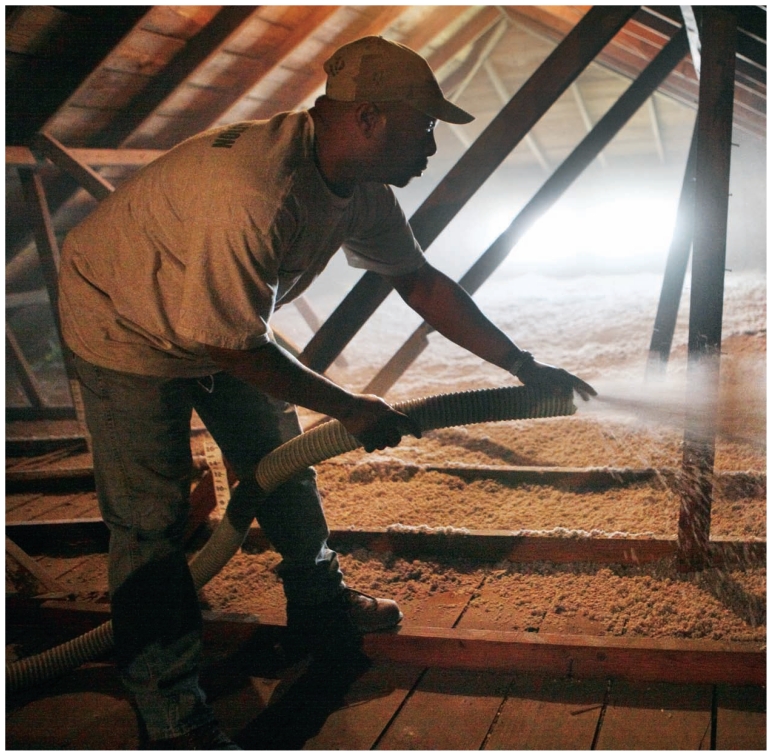
State weatherization programs got a financial boost from the federal stimulus package, but the sudden influx of cash and short time frame to spend it have raised concerns about the safety and quality of the work.

## References

[1] DOE (2008). Energy Efficiency Trends in Residential and Commercial Buildings.

[b2-ehp-119-a76] 2This figure is the sum of annual allocations listed on the DOE’s Weatherization Assistance Program website, available: http://tinyurl.com/2bv3324 [accessed 11 Jan 2011].

[b3-ehp-119-a76] 3This figure is the average of figures listed on the DOE’s Weatherization Assistance Program Allocation Formula website, available: http://tinyurl.com/4o2ca93 [accessed 11 Jan 2011].

[4] DOE Weatherization and Intergovernmental Program—About [website].

[5] DOE (2010). Audit Report. The State of Illinois Weatherization Assistance Program.

[6] Vajani M (2005). Unintentional non-fire-related carbon monoxide exposures in the United States, 2001–2003. MMWR.

[7] Texas Department of Housing and Community Affairs (2010). Monitoring Review of Nueces County Community Action Agency Weatherization Assistance Program.

[8] AP (2010). Home Mold Pops Up with Improper Weatherization. Juneau Empire, Alaska Associated Press Section, online edition.

[9] WHO (2009). WHO Guidelines for Indoor Air Quality: Dampness and Mould.

[10] Friedman GH (2009). Memorandum for the Assistant Secretary for Energy Efficiency and Renewable Energy. Audit Report. Management Alert on the Department’s Monitoring of the Weatherization Assistance Program in the State of Illinois.

[11] ASHRAE (2010). Ventilation and Acceptable Indoor Air Quality in Low-Rise Residential Buildings. Standard 62.2-2010.

[12] OSHA Green Job Hazards: Weather Insulating/Sealing [website].

[b13-ehp-119-a76] 13Backdrafting is a process whereby negative air pressure reverses the upward flow of combustion gases and draws them back into the home.

[14] Radon [website] http://tinyurl.com/y5f26n.

[15] EPA Indoor Air Quality in Homes/Residences. Controlling Moisture [website].

[16] Duct Sealing [website] http://tinyurl.com/2o23kh.

[17] DOE Energy Savers—Crawl Space Insulation [website].

[18] EPA Spray Polyurethane Foam [website].

[19] Han H (2010). Acute 1,2,3-trichloropane poisoning: a case report and literature review. Basic Clin Pharmacol Toxicol.

[20] Tardiff RG, Carson ML (2010). Derivation of a reference dose and drinking water equivalent level for 1,2,3-trichloropropane. Food Chem Toxicol.

[21] Sarathy V (2010). Degradation of 1,2,3-trichloropropane (TCP): hydrolysis, elimination, and reduction by iron and zinc. Environ Sci Technol.

[22] DOE Residential Retrofit Guidelines Project Overview—Workforce Guidelines for Home Energy Upgrades [website]. http://tinyurl.com/4aceted.

[23] EPA Indoor Air Quality in Homes—Healthy Indoor Environment Protocols for Home Energy Upgrades. http://tinyurl.com/6huco7x.

