# Erratum

**Published:** 2011-04

The February 2011 article “Avoiding Health Pitfalls of Home Energy-Efficiency Retrofits” [Environ Health Perspect 119:A76–A79 (2011)] incorrectly listed 1,2,3-trichloropropane (TCP) as a flame retardant used in spray polyurethane foams. The correct compound should have been tris(1-chloro-2-propyl) phosphate (TCPP).

*EHP* regrets the error.

